# Radiotherapy for parotid IgG4‐related disease

**DOI:** 10.1002/jmrs.304

**Published:** 2018-09-09

**Authors:** Daniel E. Roos, Marcus V. Dreosti, Craig L. James, Pravin Hissaria

**Affiliations:** ^1^ Department of Radiation Oncology Royal Adelaide Hospital and University of Adelaide, School of Medicine Adelaide South Australia Australia; ^2^ GenesisCare Adelaide South Australia Australia; ^3^ Adelaide Pathology Partners Adelaide South Australia Australia; ^4^ Clinical Immunology and Allergy Department Royal Adelaide Hospital and SA Pathology Adelaide South Australia Australia

**Keywords:** corticosteroids, IgG4‐related disease, Kimura's disease, parotid, radiotherapy

## Abstract

We describe the use of radiotherapy for parotid IgG4‐related disease (IgG4‐RD), initially misdiagnosed as Kimura's disease, with sustained good partial response in a 37‐year‐old male. To the best of our knowledge, this is the first reported case of radiation for extra‐orbital IgG4‐RD, albeit inadvertently.

## Introduction

IgG4‐related disease (IgG4‐RD) is a benign, chronic, multi‐organ disorder with fibro‐inflammatory infiltrate on histopathology, and heterogeneous clinical features depending on the site(s) of involvement. Its pathogenesis is uncertain, with both autoimmune and allergic characteristics. First described in 2003 by Kamisawa and Nakajima, the entity unifies many site‐specific diseases previously considered distinct (e.g. Riedel's thyroiditis, Kuttner's tumour of the submandibular salivary gland, autoimmune pancreatitis). The onset is typically subacute with gradual development of tumour‐like masses detected on physical examination or on imaging, in the absence of systemic symptoms. It is characterised by elevated serum IgG4 levels and IgG4‐expressing plasma cells in affected tissues, but neither are specific. Instead, careful correlation of pathology, clinical, serologic and imaging findings is required to make the diagnosis, and confusion with other disease entities, including Kimura's disease (see below) is common. IgG4‐RD responds to many systemic agents, notably corticosteroids and other immunosuppressants (e.g. methotrexate, azathioprine, cyclosporine), but relapse is frequent. More recently, targeted therapies such as the CD20 (B‐cell) monoclonal antibody rituximab, T‐cell‐depleting agents and also various immuno‐stimulatory antibodies have shown great promise.^1^ However, as far as we are aware, there have been no reports on the use of radiotherapy (RT) for any site other than the orbit since this disease entity was established.

## Case Report

**Figure 1:**
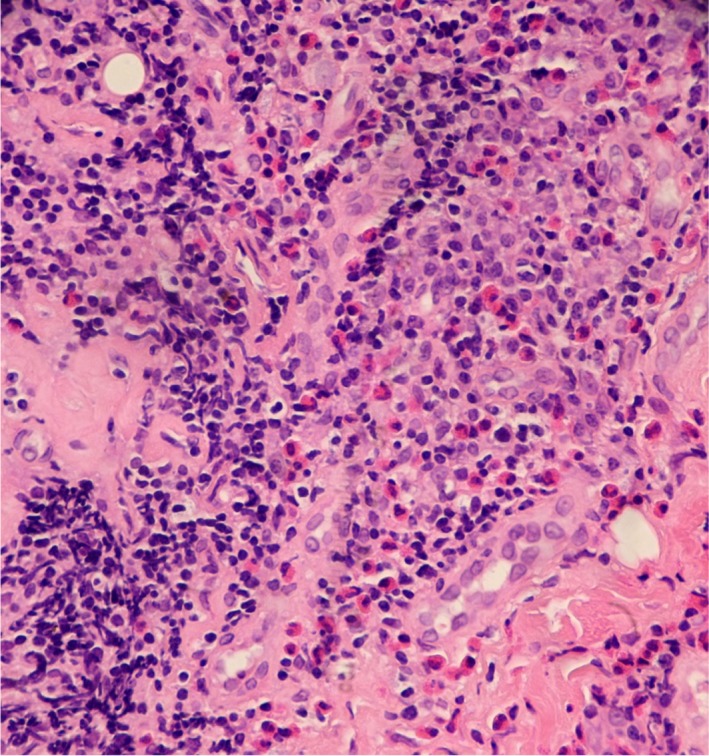
High‐power photomicrograph of left parotid biopsy (H and E × 200), showing a diffuse polymorphous inflammatory infiltrate of lymphocytes, histiocytes, eosinophils and plasma cells, together with focal stromal hyaline fibrosis.

A 37‐year‐old Burmese male was referred by his dermatologist for a RT opinion in June 2011 with left‐sided facial swelling of approximately 2 year's duration and associated local and upper body itch. His past medical history was unremarkable except for well‐controlled chronic Hepatitis B, managed with entecavir 0.5 mg daily. Serum eosinophil count (a marker of inflammatory/allergic response) was 2.6 × 10^9^/L (0.02–0.50). Fine needle aspiration cytology and subsequent skin punch biopsy in early 2010 had shown prominent eosinophil infiltrates including eosinophil micro‐abscess formation in association with lymphoid tissue, lymphoid follicles, proliferating post‐capillary venules and variable fibrosis. Plasma cells were present, although not dominant, and there was no storiform‐patterned fibrosis or phlebitis. Some vessels had hyaline thickened walls. No malignant transformation, granulomatous component, tissue parasites or significant neutrophil presence was identified (Fig. 1). The lesion site, depth of inflammatory changes with lymphoid follicles and frequent eosinophils, peripheral eosinophilia and Asian background were all consistent with the diagnosis of Kimura's disease (see below).^2^

**Figure 2:**
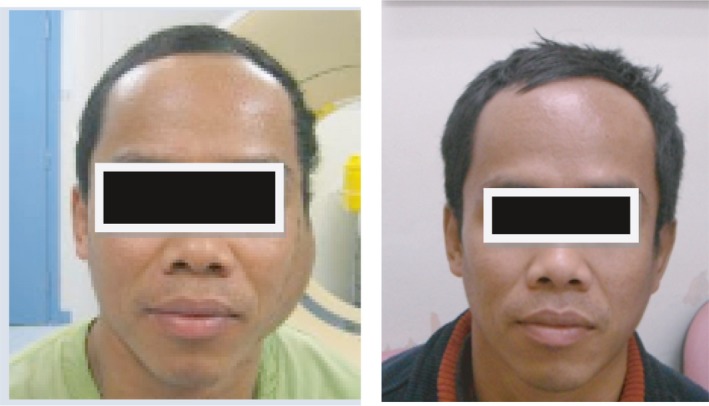
Before and 3 months after radiotherapy demonstrating excellent clinical response.

Examination revealed a firm soft tissue mass in the left pre‐ and post‐auricular region (Fig. 2). It measured 90 × 60 mm radially, 10 mm proud and the overlying skin was thickened, although not fixed to underlying structures.

**Figure 3:**
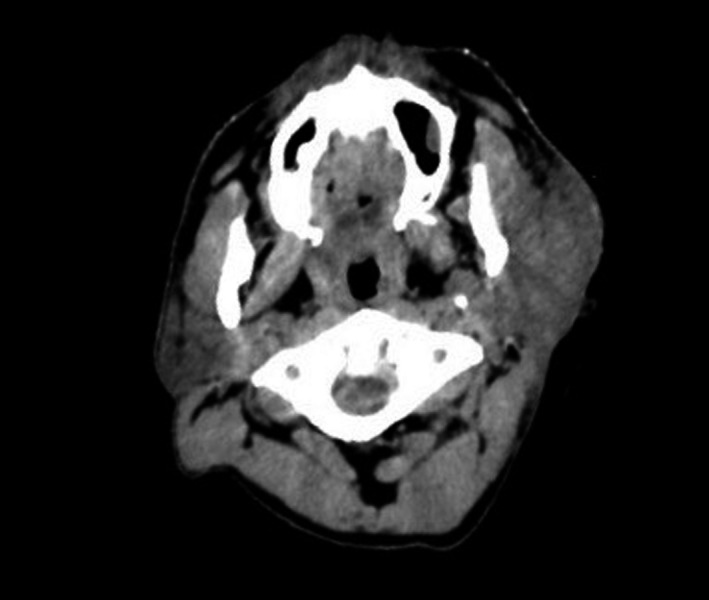
Axial planning CT showing diffuse infiltration of the left parotid and subcutaneous soft tissues (anatomical distortion due to rotated head position for electron treatment).

CT scan of the head demonstrated diffuse infiltration of the left peri‐auricular skin, subcutaneous soft tissues and parotid of maximum dimensions 90 × 90 × 35 mm but no other masses or lymphadenopathy (Fig. 3).

His initial treatment had included doxycycline with little effect, then oral prednisolone, but this was poorly tolerated and the mass recurred on weaning. After discussing alternative options, the patient consented to RT. His head and shoulders were immobilised with a thermoplastic mask for planning CT, his ear was packed with wet gauze, and the whole region was covered with 10 mm bolus. The planning target volume was defined by a 2‐cm margin on palpable and radiological disease and he received 30 Gy in 15 fractions daily over 3 weeks using a direct 16‐MeV electron field.

The treatment course was uncomplicated. By 3 months, the mass had progressively shrunk to 25 × 15 mm, 3 mm proud (Fig. 2). However, from 1 year after RT, he again required intermittent courses of prednisolone for recurrent mild left parotid swelling (much less than prior to treatment) with associated itch. From 3&frac12; years after RT, *right* parotid swelling became the dominant problem, with bilateral parotid biopsies in August 2016 confirming active disease on both sides consistent with the original diagnosis (independently via a second pathologist). Repeat CT head/neck/chest/abdomen at that time (and previously in 2013), showed involvement of the parotid regions only. During follow‐up, he also had opinions from medical oncology, haematology, ENT and immunology specialists. Immunosuppressants were considered, but relatively contraindicated because of his history of Hepatitis B.

In May 2017, his immunologist raised the possibility of IgG4‐RD as an alternative diagnosis. Further immunostaining of the 2016 specimens revealed an IgG4/IgG ratio of 59%, with up to 109 IgG4 labelled plasma cells per high power field, thereby satisfying the criteria for ‘highly suggestive’ of salivary IgG4‐RD, namely >40% and >100%, respectively.^3^ The serum IgG4 was also mildly elevated at 1.04 g/L (0.12–0.96).

At review by his immunologist 6 years after RT, his facial appearance was essentially a mirror image of the original presentation, with prominence of the right, but not the treated left parotid and there was a suspicious new skin lesion on the abdomen. Accordingly, the decision was finally taken (reluctantly) by his immunologist to commence cyclosporine 100 mg BD, in order to minimise the need for further courses of steroids. This led to rapid response of all disease sites. RT remains an option for the right parotid region (or elsewhere) if needed in the future.

## Discussion

This case study demonstrates that intermediate dose RT can provide sustained local cosmetic benefit for parotid IgG4‐RD (albeit inadvertently here for the wrong diagnosis). It also demonstrates that interpretation of IgG4‐RD pathology can be challenging, particularly because histologic characteristics are heterogeneous at different sites. The chosen dose of 30 Gy in 15 fractions was based upon the original diagnosis of Kimura's disease, a benign inflammatory disorder of uncertain aetiology, possibly an aberrant immune reaction to an unknown antigenic stimulus. It is rare except in the Asian population, but sharing many histopathological features with IgG4‐RD which partly explains the diagnostic confusion here. There are anecdotes of Kimura's disease associated with a distant history of arthropod bite and, interestingly, this patient did report an insect bite on his left ear while working in the Burmese rice fields some years previously. Indeed, this was one of the features which led his referring dermatologist to the initial clinical diagnosis. Kimura's disease manifests with solitary or multiple subcutaneous nodules or masses often accompanied by regional lymphadenopathy and/or salivary gland hypertrophy, most commonly affecting the head and neck area, particularly the parotid and cervical regions. It typically presents in the third decade of life, with a marked male predominance. Systemically, there is usually marked eosinophilia and elevated serum immunoglobulin E (IgE) levels. The disease course can be chronic and relapsing and results in significant morbidity as the slowly enlarging masses become painful and locally infiltrative, but malignant transformation has not been observed. Histological examination characteristically reveals subcutaneous mixed inflammatory infiltrates including reactive lymphoid follicles and frequent eosinophils. Proliferating post‐capillary venules are lined by generally flattened endothelial cells, mast cells are increased whilst there may be eosinophil abscesses, eosinophil‐rich granulomas, follicular lysis, polykaryocytes, IgE expression and fibrosis in older lesions.^2^

RT has been used for Kimura's disease, reported doses ranging from 13 to 56 Gy, with no dose‐response beyond 26–30 Gy in conventional fractionation.^4^ There are limited data on RT for orbital IgG4‐RD using doses similar to those in the past for orbital ‘pseudotumour’ (around 20 Gy),^5^ but to the best of our knowledge, no previous reports on RT for extra‐orbital involvement. It is possible that a dose lower than 30 Gy would have been effective here also.

In summary, this case demonstrates that IgG4‐RD can constitute a clinical and pathological diagnostic dilemma, as none of the features are unique. The possibility needs to be entertained in the setting of slowly enlarging, histologically benign mass(es), particularly in the head and neck region. Similar to competing non‐malignant diagnoses such as Kimura's disease, IgG4‐RD is radio‐responsive. This was already known for orbital disease, but we have demonstrated radio‐sensitivity (and sustained partial response) also for IgG4‐RD of the parotid region. Given the availability of many systemic agents, and that RT for non‐malignant diseases should always be undertaken with caution, we are not advocating that RT be high on the list of treatment options for IgG4‐RD, rather that it is a *potential* option if needed, particularly for localised involvement.

## Ethics statement

This case study was approved by the Royal Adelaide Hospital Research Ethics Committee and the patient gave written informed consent.

## Conflict of Interest

The authors declare no conflict of interest.
